# Efficient 2,3-Butanediol Production from Ethanol by a Modified Four-Enzyme Synthetic Biosystem

**DOI:** 10.3390/molecules29163934

**Published:** 2024-08-20

**Authors:** Jiming Zhang, Hui Lin, Chaosong Zheng, Bin Yang, Miao Liang, Yi Lin, Liaoyuan Zhang

**Affiliations:** 1 College of Chemical Engineering, Huaqiao University, Xiamen 361021, China; jemeza@126.com; 2 Institute of Edible Fungi, Fujian Academy of Agricultural Sciences, Fuzhou 350012, China; 2170517002@fafu.edu.cn; 3 College of Life Sciences, Fujian Agriculture and Forestry University, Fuzhou 350002, China; 52305043027@fafu.edu.cn (C.Z.); lmiaojy@163.com (M.L.)

**Keywords:** ethanol upgrading, semi-rational design, catalytic performance, substrate tolerance, cell-free multienzyme catalysis

## Abstract

2,3-butanediol (2,3-BD) is a versatile bio-based platform chemical. An artificial four-enzyme synthetic biosystem composed of ethanol dehydrogenase, NADH oxidase, formolase and 2,3-butanediol dehydrogenase was designed for upgrading ethanol to 2,3-BD in our previous study. However, a key challenge in developing in vitro enzymatic systems for 2,3-BD synthesis is the relatively sluggish catalytic efficiency of formolase, which catalyzes the rate-limiting step in such systems. Herein, this study reports how engineering the tunnel and substrate binding pocket of FLS improved its catalytic performance. A series of single-point and combinatorial variants were successfully obtained which displayed both higher catalytic efficiency and better substrate tolerance than wild-type FLS. Subsequently, a cell-free biosystem based on the FLS:I28V/L482E enzyme was implemented for upgrading ethanol to 2,3-BD. Ultimately, this system achieved efficient production of 2,3-BD from ethanol by the fed-batch method, reaching a concentration of 1.39 M (124.83 g/L) of the product and providing both excellent productivity and yield values of 5.94 g/L/h and 92.7%, respectively. Taken together, this modified enzymatic catalysis system provides a highly promising alternative approach for sustainable and cost-competitive production of 2,3-BD.

## 1. Introduction

2,3-butanediol (2,3-BD), one of the more promising platform chemicals, is extensively used to synthesize various usable chemicals and versatile materials [1,2,3,4,5,6]. Commercially available 2,3-BD is manufactured mainly from petroleum feedstocks by chemical processes. However, such chemosynthetic methods generally require harsh reaction conditions and a complicated synthesis process and thus trigger environmental hazards [7]. Instead, biorefinery of 2,3-BD has attracted considerable attention, as it fulfills the criteria of “environmentally benign chemistry” and “green chemistry” [8,9].

Currently, three biotechnological strategies have been developed to synthesize 2,3-BD by microbial fermentation, whole-cell catalysis and enzymatic catalysis [10,11,12,13,14]. Many efforts, including strain screening, condition optimization and development of novel fermentation strategies and engineering strains, have been made for 2,3-BD production by microbial fermentation, resulting in excellent product concentration, high productivity and high yield [1,3,4,15]. However, by-products (e.g., ethanol, acetic acid and lactate, etc.) are inevitably produced during the 2,3-BD fermentative process, making the preparation of 2,3-BD from fermentation broth extremely difficult and economically costly [16,17]. Similarly, whole-cell catalysis is restrained by complex processing steps [18]. Moreover, the cellular wall/membrane poses a constraint and thus causes mass transfer limitations of substrates and products during the catalysis process [19]. By contrast, enzymatic catalysis exhibits unique advantages, such as fast reaction rates, high product yields, flexible process optimization and product purification, etc. [20,21].

Strikingly, an in vitro biosynthetic platform which was driven by crude lysates of engineered *Escherichia coli* cells was created to produce 82.0 g/L of meso-2,3-BD by using glucose as a substrate, resulting in productivity and theoretical yield values of 2.7 g/L/h and 64.0%, respectively [10]. Recently, 186.7 g/L of acetoin, the precursor of 2,3-BD, was efficiently produced from pyruvate by a dual-enzyme cascade system composed of α-acetolactate synthetase (ALS) and α-acetolactate decarboxylase (ALDC), and theoretical yield and productivity values of 94.3% and 15.56 g/L/h were obtained [22]. Unfortunately, in the above-mentioned system there is obvious carbon loss due to a decarboxylation reaction, and pyruvate is a relatively expensive substrate. A similar system coupled carbonyl reductase and glucose dehydrogenase to produce 12.2 g/L acetoin from 14.3 g/L diacetyl [23]. Nevertheless, the high toxicity of diacetyl to enzymes reduces the competitiveness of this method. Taken together, producing 2,3-BD via enzymatic catalysis remains a challenge.

The emergence of C-C bond-forming enzymes (e.g., formolase, pyruvate decarboxylase, E1 subunit of α-ketoglutarate dehydrogenase, etc.) opens exciting opportunities for producing acetoin from acetaldehyde [24,25,26,27]. This kind of enzymatic reaction is favorable due to the following advantages: (i) it fulfills the principle of the atom economy because there is no carbon loss; (ii) it is economically viable because acetaldehyde is a more inexpensive feedstock compared to diacetyl and pyruvate; and (iii) acetaldehyde is extremely abundant due to the expanding biomass-based ethanol industry [28]. In particular, formolase (FLS) has become a fast-rising star due to it having the best catalytic activity for carboligation of acetaldehyde among the tested enzymes [25,27].

**Figure 1:**
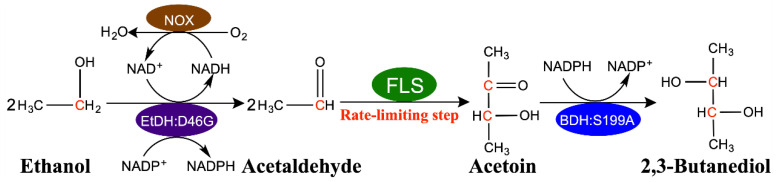
Schematic of the four-enzyme synthetic biosystem for upgrading ethanol to 2,3-butanediol. During the reaction process, the NADH produced was rapidly recycled back to NAD^+^ via the H_2_O-forming NOX, while the NADPH generated was used to produce 2,3-butanediol from acetoin.

In our previous work, the proof-of-concept biosynthesis of 2,3-butanediol from ethanol was conducted with a four-enzyme synthetic biosystem composed of ethanol dehydrogenase (EtDH:D46G), NADH oxidase (NOX), formolase (FLS) and 2,3-butanediol dehydrogenase (BDH:S199A) (Figure 1). Briefly, the EtDH:D46G was responsible for the oxidation of ethanol to acetaldehyde, along with the simultaneous utilization of NAD^+^ and NADP^+^ as coenzymes. Subsequently, FLS was used to irreversibly catalyze acetaldehyde into acetoin. Finally, acetoin was converted into 2,3-BD by NADPH-dependent BDH:S199A. In this system, we designed a dual cofactor regulatory system to maintain NAD(P)H balance and recycling without the buildup of additional reducing equivalents. During the reaction process, the NADH produced was rapidly recycled back to NAD^+^ via the H_2_O-forming NOX, while the NADPH generated was used to produce 2,3-butanediol from acetoin (Figure 1) [27]. Unfortunately, the relatively sluggish catalytic efficiency of FLS was deemed as the rate-limiting step of this system, thus hindering its foregrounding in industrial applications.

Over the past few decades, protein engineering has been frequently employed to enhance the catalytic performance of natural enzymes. Generally, the major strategies used include directed evolution technology, semi-rational or rational design, and de novo design [29,30,31]. Among these, semi-rational design that mutates hotspot residues according to prior structural or functional information showed its validity in that it is more likely to achieve positive results, and simultaneously reduces work intensity compared with random mutagenesis [32]. In the present study, a structure-guided semi-rational design was followed to improve the catalytic performance of FLS by simultaneously tailoring candidate “hotspots” of the binding pocket and the entrance tunnel. Finally, high-level 2,3-BD was smoothly synthesized from ethanol in a modified cell-free multienzyme system.

## 2. Results and Discussion

### 2.1. Selection of Mutational Residues

**Figure 2:**
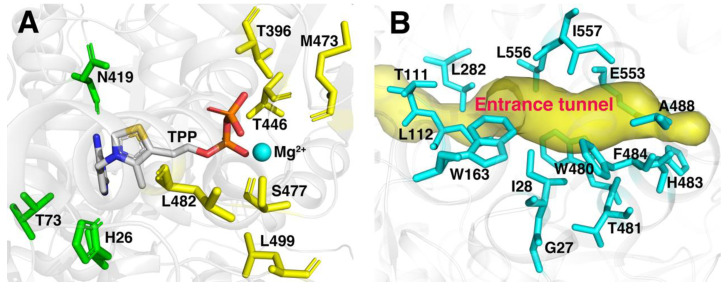
Hotspots of FLS for mutagenesis in the binding pocket (**A**) and the entrance tunnel (**B**).

In our previous study, six residues (T396, T446, M473, S477, L482 and L499) regarded as mutational hotspots in FLS (Figure 2A) were altered by site-saturated mutagenesis and assessed for enzyme activity using acetaldehyde as a substrate. The variant L482S in FLS showed higher catalytic efficiency and yield for the conversion of acetaldehyde to acetoin under low substrate concentration in our previous study [27]. However, the reduction in product yield with the increase in the substrate concentration by FLS:L482S required further improvement for its catalytic efficiency and substrate tolerance. FLS belongs to the group of thiamine diphosphate (TPP)-dependent enzymes derived from benzaldehyde lyase (BAL) in *Pseudomonas fluorescens biovar* I, which were initially designed to carry out a formose reaction [26]. Acetaldehyde as a non-natural substrate of the FLS enzyme may require a more suitable size and shape of the substrate binding pocket and entrance tunnel. Extensive success stories also demonstrated that reshaping the substrate binding pocket has already been shown to tremendously improve enzyme catalytic efficiency for non-natural substrates [33,34]. Additionally, the enzyme entrance tunnels connecting the environment with the binding pocket are also deemed to be crucial for the catalytic properties of enzymes [35,36]. It is worth emphasizing that acetaldehyde is a highly reactive and difficult-to-tame chemical. Consequently, the particular size and polarity of the entrance tunnel may influence the access of the substrate or the release of the product. Herein, the substrate binding pocket and access tunnel in FLS were analyzed with PyMol software (http://www.pymol.org) and CAVER 3.0 software (http://www.caver.cz). As shown in Figure 2, 23 residues located around the substrate binding pocket (H26, T73, T396, N419, T446, M473, S477, L482 and L499) and entrance tunnel (G27, I28, T111, L112, W163, L282, W480, T481, H483, F484, A488, E553, L556 and I557) of FLS were observed. Aside from 6 residues (T396, T446, M473, S477, L482 and L499) investigated as hotspots by HotSpot Wizard 2.0 server in our previous studies, the remaining 17 residues were selected as candidate sites to evaluate the effects on its catalytic efficiency and substrate tolerance. Additionally, the residue L482 was also regarded as key site for performing combinatorial mutations in this study due to its positive effect on enzyme activity [27].

### 2.2. Single-Point and Combinatorial Mutation of FLS

**Figure 3:**
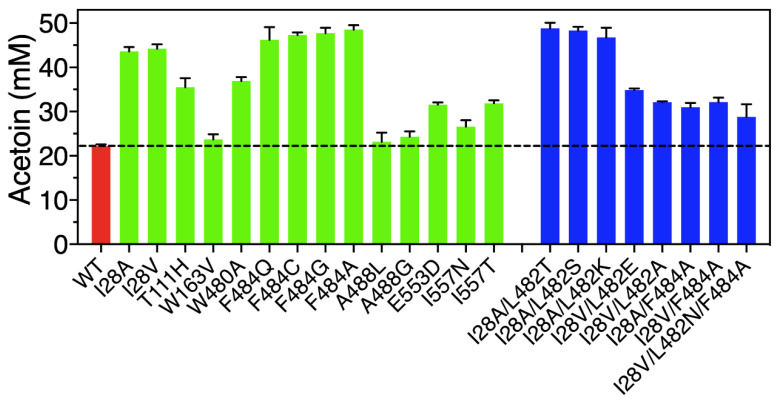
Acetoin production from acetaldehyde via whole-cell catalysis by wild-type FLS and its variants. The screening was performed in a whole-cell catalytic system at 30 °C for 6 h (100 mM phosphate buffer, 40 g/L wet cell weight, 100 mM acetaldehyde, 0.1 mM TPP, 1 mM Mg^2+^, pH 8.0).

For high-efficiency screening of positive variants, we employed a high-throughput approach to determine the yield of acetoin produced by whole-cell catalysis combined with a VP color reaction, which was assayed with a microplate reader at 520 nm. After assays of 1700 variants, 8 out of 17 sites in the FLS enzyme exhibited beneficial effects on enzyme activity for the conversion of acetaldehyde to acetoin (Figure 3). Among them, altering the 28 and 484 sites located in the entrance tunnel of FLS showed obvious improved acetoin yields. Two variants, I28A and I28V, in the FLS enzyme could produce higher acetoin concentrations of 43.58 and 44.21 mM with yields of 87.16% and 88.42% from 100 mM acetaldehyde, respectively. More encouragingly, 46.20, 47.31, 47.70 and 48.51 mM of acetoin were obtained using the variants F484Q, F484C, F484G and F484A, which represented acetoin yields of 92.40%, 94.62%, 95.40% and 97.02% of the theoretical yield, respectively, while only 22.16 mM of acetoin with a yield of 44.32% could be obtained from 100 mM acetaldehyde by wild-type FLS. In addition, the variants T111H, W163V, W480A, A488L, A488G, E553D, I557N and I557T also showed different positive effects on acetoin production, with concentration ranges from 23.14 to 36.92 mM (Figure 3). The above results indicate that altering the sites surrounding the substrate entrance tunnel exhibited good potential for improving FLS activity. In particular, the variants I28A, I28V, F484G and F484A showed excellent acetoin yields. On the contrary, mutating the H26, T73 and N419 sites located in the substrate binding pocket resulted in an obvious reduction in the acetoin yields (Figure S1), implying that the three sites played important roles for maintaining FLS activity for acetoin production from acetaldehyde.

Based on the above results, combinatorial mutagenesis of the sites I28 and F484 plus L482 was systematically implemented to investigate their synergistic interactions. Seven double mutants and one triple mutant were developed and exhibited better catalytic performance than wild-type FLS (Figure 3). Among them, 48.81, 48.31 and 46.75 mM of acetoin from 100 mM acetaldehyde were obtained by double mutants of I28A/L482T, I28A/L482S and I28A/L482K, respectively, which represented obvious improvements of 2.20-, 2.18- and 2.11-fold relative to wild-type FLS (22.16 mM).

### 2.3. Acetaldehyde Tolerance Evaluation of FLS and Its Variants

**Figure 4:**
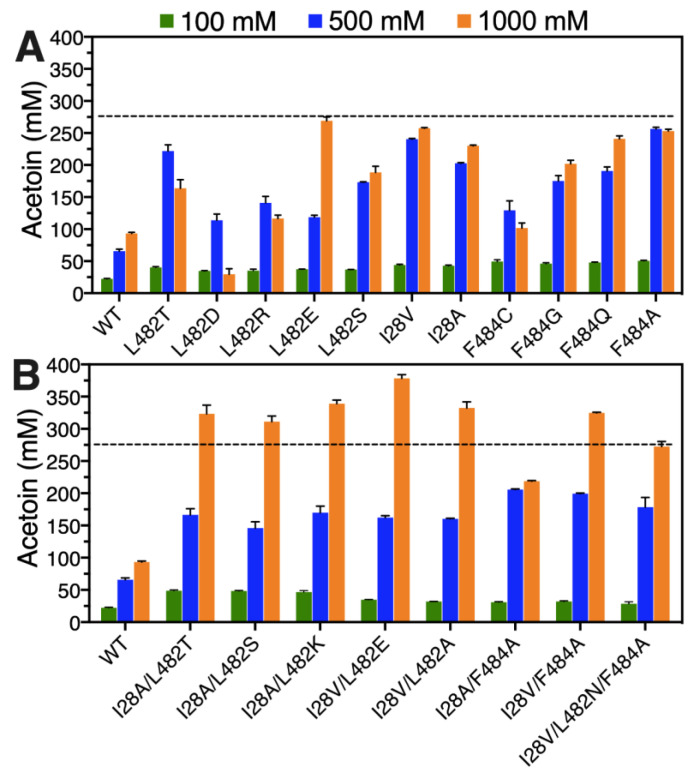
Acetaldehyde tolerance evaluation of recombinant *E. coli* cells harboring wild-type FLS and its single-site mutants (**A**) or combinatorial mutants (**B**). The whole-cell biocatalysis was conducted at 30 °C for 6 h in reaction mixtures (100 mM phosphate buffer, 40 g/L wet cell pellets, 100–1000 mM acetaldehyde, 0.1 mM TPP, 1 mM Mg^2+^, pH 8.0).

The effects of different substrate concentrations (100, 500 and 1000 mM) on the conversion of acetaldehyde to acetoin were firstly investigated via whole-cell catalysis by wild FLS and its beneficial variants. For single-point substitutions, the recombinant *E. coli* cells containing the variants F484A, I28V and L482T exhibited excellent yields of 99.69%, 96.21% and 88.76%, respectively, in the presence of acetaldehyde with 500 mM, and the corresponding acetoin concentrations reached 249.23, 240.52 and 221.89 mM (Figure 4A). When a substrate concentration of 1000 mM was used, the acetoin yields for recombinant *E. coli* cells harboring all the beneficial variants except the L482E variant showed significant decreases (Figure 4A), suggesting that a high-concentration substrate may inhibit the enzyme activity or result in enzyme denaturation. However, an encouraging phenomenon shown in Figure 4A was that the acetoin concentration was continuously increased with increasing substrate concentration by recombinant *E. coli* cells harboring the L482E variant. A maximum acetoin concentration of 268.99 mM with a yield of 53.80% from 1000 mM acetaldehyde could be achieved by the L482E variant (Figure 4A), implying that altering leucine to glutamic acid at site 482 of the FLS enzyme could efficiently improve substrate tolerance. For all multiple-point substitutions, the acetoin titer showed a rising tendency with increasing substrate concentration by whole-cell catalysis, as shown in Figure 4B. Except for the variant I28A/F484A, a final acetoin concentration in the range of 272.72 to 378.34 mM could be achieved from 1000 mM acetaldehyde by these beneficial combinatorial mutants, which was significantly higher than the highest acetoin titer (268.99 mM corresponding to recombinant *E. coli* cells harboring the L482E variant) that was produced by single-point substitution. A maximum acetoin concentration of 378.34 mM with a yield of 75.67% was obtained via whole-cell catalysis by the variant I28V/L482E, which presented a 4.05-fold increment in acetoin concentration compared with recombinant *E. coli* cells harboring wild-type FLS.

**Figure 5:**
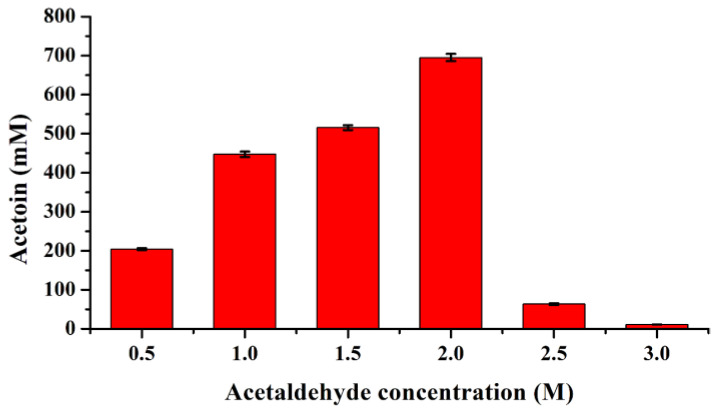
Acetaldehyde tolerance assay of the purified I28V/L482E variant for acetoin production. The reaction was conducted at 30 °C for 6 h in 0.5 mL solution containing 100 mM phosphate buffer (pH 8.0), 100 μg purified enzyme, 0.5–3.0 M acetaldehyde, 0.1 mM TPP and 1 mM Mg^2+^.

To further investigate its robustness, the I28V/L482E variant was purified and used to exactly assess its substrate tolerance (Figure S2). The results showed that 447.34 and 695.05 mM acetoin with yields of 89.47% and 69.51% could be obtained in the presence of 1.0 and 2.0 M acetaldehyde, respectively, by the purified I28V/L482E variant (Figure 5), while a sharp reduction in acetoin yield could be observed when an initial acetaldehyde concentration of over 2.0 M was used, which was mainly attributed to enzyme denaturation (Figure S3). The above results indicate that the double mutant I28V/L482E was the most promising variant that displayed the best catalytic performance as well as excellent substrate tolerance; thus, FLS:I28V/L482E was used in the subsequent cell-free multienzyme system.

### 2.4. Kinetic Parameters

The kinetic parameters of wild-type FLS and its four beneficial variants were determined by using purified enzymes (Figure S4). As shown in Table 1, *k*_cat_ values of 3.33, 0.49, 1.74 and 8.76 s^−1^ when using acetaldehyde as a substrate could be achieved by the variants I28V, L482E, F484A and I28V/L482E, respectively, which showed 7.40-, 1.09-, 3.87- and 19.47-fold increases relative to wild-type FLS (0.45 s^−1^). Relatively low *K*_m_ values for the L482E and F484A variants could be observed when compared with that for the wild-type enzyme and resulted in *k*_cat_/*K_m_* values of 9.66 and 32.56 s^−1^ M^−1^, respectively (Table 1), while the *K*_m_ values of 81.95 and 375.55 mM for the variants I28V and I28V/L482E were higher than those for wild-type FLS, L482E and F484A, which may be attributed to the increased pocket sizes in both variants, which were further analyzed in subsequent structure modeling in silico. Considering the catalytic performance and substrate tolerance, the variant I28V/L482E was chosen to form the cell-free pathway from ethanol to 2,3-butanediol.

### 2.5. In Silico Structure Analysis of Wild FLS and Its Variant I28V/L482E

**Figure 6:**
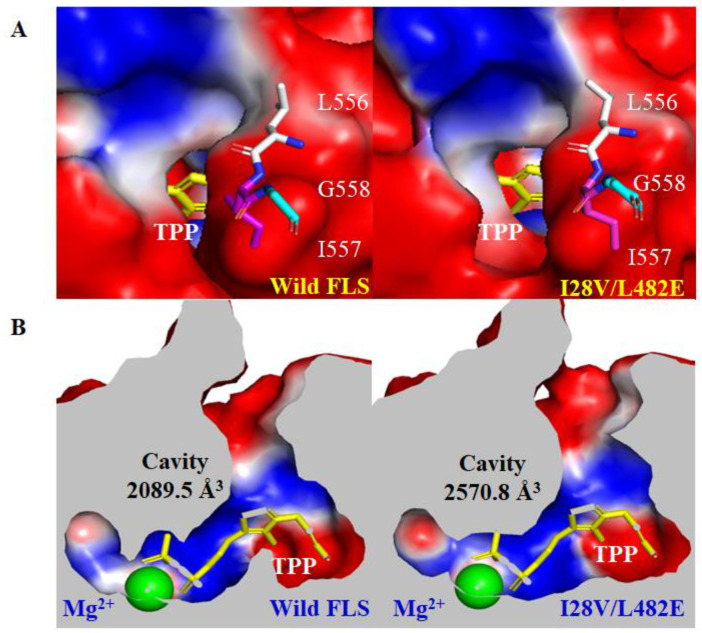
The comparison of the entrances to substrate tunnels (**A**) and active cavities (**B**) between wild FLS and its variant I28V/L482E using Caver 3.0 and Pymol 2.0.

The 3D structure of wild FLS (ID: 4QQ8) was obtained from the RCSB PDB database, and its variant I28V/L482E was modeled using AlphaFold 2.0. Molecular dynamics (MD). Simulations of both the structure models were performed for 50 ns in 10 Å SPCE with Gromacs 2020.4 with the force field amber99sb-ildn. Both the structure models were randomly selected at 45 ns to analyze the changes in the substrate entrance tunnel and active cavity with CAVER 3.0 software after systematic stability was achieved. The results revealed that the variant I28V/L482E had an increased bottleneck radius and throughput as well as reduced length and curvature of the substrate entrance tunnel when compared with wild FLS (Figure 5S and Table S1). In addition, the entrance to the substrate tunnel in wild FLS is partially covered on its structure surface by its C-terminal helix (556L-557I), while the variant I28V/L482E resulted in the movement of the C-terminal helix and thus more exposure of the entrance to substrate tunnel (Figure 6A). Previous studies indicated that the length and movement of a C-terminal helix in C-C bond-forming enzymes (e.g., formolase, pyruvate decarboxylase, E1 subunit of α-ketoglutarate dehydrogenase, etc.) were important for catalytic activity, cofactor binding and substrate specificity [37,38,39]. Therefore, these changes in the structure of the variant I28V/L482E may favor the access of acetaldehyde and the release of the product acetoin, and thus lead to higher catalytic efficiency. Furthermore, the comparison of the active cavities between wild FLS and its variant I28V/L482E showed obvious changes in binding pocket size (Figure 6B). The calculated cavity size (2570.8 A^3^) in the I28V/L482E variant was much bigger than that in wild-type FLS (2089.5 245 A^3^), which corresponded to a higher *K*_m_ value and a lower affinity to the substrate acetaldehyde as assayed for kinetic parameters (Figure 6B and Table 1).

**Figure 7:**
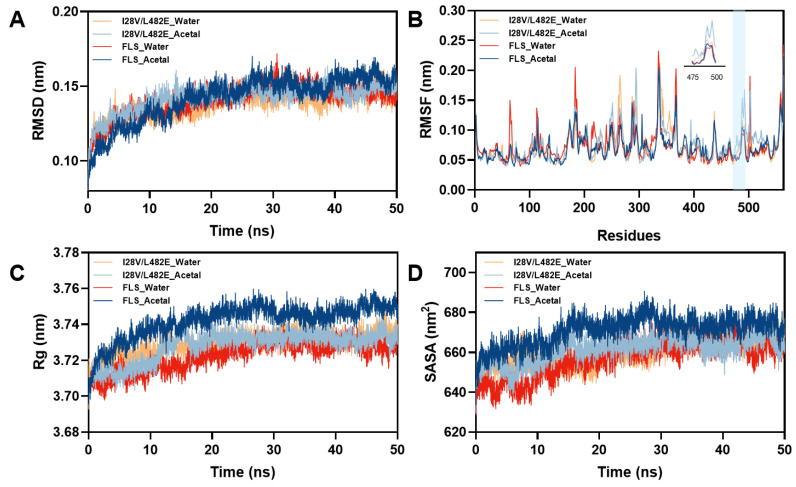
Overall (**A**), local structure (**B**), radius of gyration (**C**) and solvation (**D**) changes in wild FLS and its variant I28V/L482E in pure water and 1 M acetaldehyde.

To gain insight into higher substrate tolerance, MD simulations were carried out on wild FLS and its variant I28V/L482E in acetaldehyde (1 M) and pure water. The root mean square deviation (RSMD) of the backbone atoms in wild FLS and its variant was analyzed (Figure 7A). In pure water and 1 M acetaldehyde, the RSMD values of 0.1438 Å and 0.1476 Å for the variant I28V/L482E averaged in the last 30 ns of the simulation, respectively, were smaller than those for wild FLS (0.1474 Å and 0.1529 Å), indicating that the backbones in the variant I28V/L482E possessed higher stability than those in wild FLS in pure water or 1 M acetaldehyde. The root mean square fluctuation (RSMF) values were analyzed in pure water as well as 1 M acetaldehyde. Obvious changes with increased RMSF values could be observed in two loops (N475-W480 and G490-R500) of the variant I28V/L482E in 1 M acetaldehyde (Figure 7B), which represented increased flexibility of the two loops around the substrate tunnel. Analysis of the internal H-bond and surface salt bridge numbers indicated that two H-bonds (N419-W480 and W478-T481) and one salt bridge (E553-H483) within the two loops of the variant I28V/L482E disappeared in the 1 M acetaldehyde solution compared to wild FLS in water or 1 M acetaldehyde. This finding suggested that the variant I28V/L482E eliminated unfavorable internal H-bonds and surface salt bridges, leading to flexibility modification under high acetaldehyde concentrations, which might contribute to the resistance to high substrate concentrations. Similar phenomena were observed and analyzed in *Bacillus subtilists* Lipase A (BSLA) and its variants [40,41]. Furthermore, the changes in the radius of gyration (Rg) and solvent accessible surface area (SASA) revealed a tighter structure for the variant I28V/L482E in comparison to that for wild FLS in 1 M acetaldehyde (Figure 7C,D). The total sum of internal H-bond numbers within the structure of the variant I28V/L482E increased from 915 to 920 compared to that in wild FLS during the MD simulation process in 1 M acetaldehyde. Meanwhile, total salt bridge numbers of 233 and 243 could be counted in wild FLS and its variant, respectively, in 1 M acetaldehyde. The increases in the numbers of total internal H-bonds and surface salt bridges might result in a more tight structure of the variant I28V/L482E, leading to more substrate tolerance.

### 2.6. Fed-Batch Synthesis of 2,3-Butanediol from Ethanol by a Modified Four-Enzyme Synthetic Biosystem

**Figure 8:**
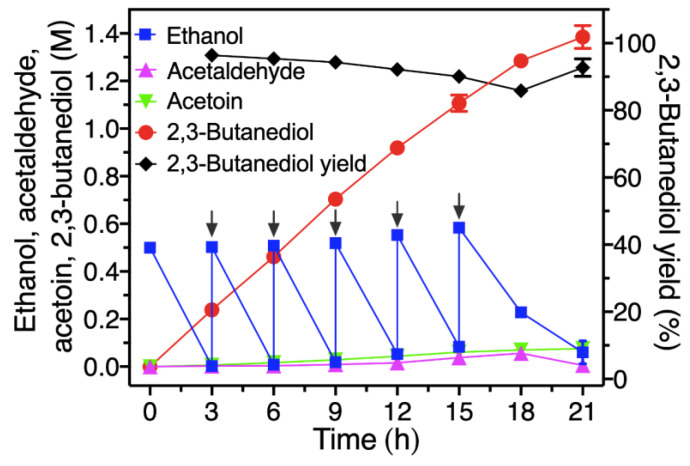
Fed-batch synthesis of 2,3-BD from ethanol by a four-enzyme synthetic biosystem. The reaction was conducted at 30 °C in HEPES buffer (50 mM, pH 8.0) containing 1 mM NAD^+^, 1 mM NADP^+^, 5 mM Mg^2+^, 1 mM DTT, 0.5 mM TPP, 20% DMSO and four crude enzymes of 2.5 mg/mL EtDH, 5 mg/mL FLS:I28V/L482E, 2.5 mg/mL BDH:S199A and 2.5 mg/mL NOX. The initial concentration of ethanol was 0.5 M, and then approximately 0.5 M ethanol was repeatedly supplemented into the reaction mixture.

Herein, the variant FLS:I28V/L482E instead of the variant FLS:L482S [27] was used to conduct the reaction from ethanol to 2,3-butanediol (Figure 1). The initial reaction mixture contained four crude enzymes (2.5 mg/mL EtDH/D46G, 5 mg/mL I28V/L482E, 2.5 mg/mL BDH:S199A and 2.5 mg/mL NOX), 1 mM NAD^+^, 1 mM NADP^+^, 5 mM Mg^2+^, 1 mM DTT, 0.5 mM TPP, 20% DMSO and 0.5 M ethanol with HEPES buffer (50 mM, pH 8.0). As shown in Figure 8, the 0.5 M ethanol in the initial reaction mixture was rapidly consumed, and converted into 0.24 M of 2,3-butanediol with a yield of 95.41% after 3 h, while the 2,3-butanediol yield was only 50.92% of its theoretical yield (0.13 M 2,3-butanediol from 0.5 M ethanol) when the mutant FLS:L482S was used to conduct the reaction in our previous study [27]. A continuous increase in 2,3-butanediol could be observed with rapid consumption of ethanol via feeding the substrate into the reaction system before 15 h. Thereafter, the rate of substrate consumption gradually slowed down, partially due to enzyme denaturation and precipitation. Ultimately, 1.39 M (124.83 g/L) of 2,3-butanediol from 3.0 M ethanol was efficiently synthesized at 21 h by the fed-batch method, with an excellent productivity of 5.94 g/L/h and a yield of 92.7%. In addition, the intermediates of acetaldehyde and acetoin were kept at low levels during the whole reaction process, and less than 0.07 M ethanol was accumulated at the end of the reaction, suggesting that the product 2,3-BD could readily be separated from the reaction solution (Figure 8).

Cell-free biomanufacturing of 2,3-butanediol and acetoin has been reported in previous studies. Among them, Jewett et al. reported a productivity of up to 2.7 g/L/h in the synthesis of 82.0 g/L 2,3-butanediol catalyzed by crude lysates of engineered *E. coli* cells from glucose [10]. Similarly, 13.52 g/L 2,3-butanediol was synthesized from cheap starch in an enzymatic reaction system containing a cocktail of lysates derived from cyanobacteria and *E. coli* [42]. Further, 186.7 g/L acetoin was obtained from pyruvate at a high productivity of 15.56 g/L/h by a powerful dual-enzyme cascade system composed of pure α-acetolactate synthetase and pure α-acetolactate decarboxylase [22]. Nevertheless, the relatively costly feedstock reduces the competitiveness of this method. Additionally, in all the above-mentioned systems there is obvious carbon loss due to decarboxylation reactions [18]. Compared with the other reported methods of in vitro enzymatic synthesis of 2,3-butanediol (Table 2), higher values of the 2,3-butanediol titer (124.83 g/L) and productivity (5.94 g/L/h), with a 92.7% theoretical yield from ethanol, were achieved in this study, which boosts the industrial feasibility of such a method because of its advantages, including the following: (i) this system really achieves zero waste of carbon atoms, enabling near 100% theoretical yield from input ethanol; (ii) it is economically viable because ethanol is cost-competitive and abundant due to the expanding biomass-based ethanol industry; and (iii) the substrate ethanol and intermediates (acetaldehyde and acetoin) were maintained at low concentrations throughout the catalytic process, which facilitates future downstream 2,3-butanediol separation. Moreover, some effective strategies, such as enzyme immobilization, self-assembly of multienzymes, industrial process optimization and mining of thermostable enzymes with excellent catalytic performance, are expected to further improve the robustness of our system and reduce the cost in future work.

## 3. Materials and Methods

### 3.1. Chemicals and Reagents

Q5 DNA polymerase and DpnI (restriction endonuclease) were obtained from NEB (Ipswich, MA, USA). Dithiothreitol (DTT), HEPES, isopropyl-*β*-D-thiogalactopyranoside (IPTG) and kanamycin were bought from Sangon Biotech Co., Ltd. (Shanghai, China). The coenzymes (Thiamine pyrophosphate (TPP), NAD^+^ and NADP^+^) were obtained from Sigma-Aldrich (Saint Louis, MO, USA). Ethanol (99%), acetaldehyde (40%), (3*S/*3*R*)-acetoin (99%) and 2,3-butanediol (99%) were obtained from Aladdin (Shanghai, China) and TCI (Tokyo, Japan), respectively.

### 3.2. Bacterial Growth, Recombinant Protein Expression and Purification

*Escherichia coli DH5α* and BL21 (DE3) were selected as host strains for the construction of plasmids and the expression of recombinant proteins, respectively. The recombinant plasmids pET28a-EtDH:D46G, pET28a-NOX, pET28a-FLS and pET28a-BDH:S199A were maintained in our laboratory, and were used for overexpression of ethanol dehydrogenase (EtDH:D46G), NADH oxidase (NOX), formolase (FLS) and 2,3-butanediol dehydrogenase (BDH:S199A), respectively [27]. For protein expression, the recombinant *E. coli* BL21 (DE3) strain was cultured in Luria–Bertani (LB) medium containing 50 μg/mL kanamycin at 37 °C, and then induced (18 °C, 24 h) by adding 0.5 mM IPTG when the OD_600_ of cell density reached 0.6~0.9. The induced cells were harvested by centrifugation and disrupted by ultrasonication for 10 min in an ice bath, and then the cell debris was discarded by centrifugation (12,000× *g*, 20 min) at 4 °C to obtain the crude enzyme. Subsequently, the crude enzyme was purified using a HisTrap Ni-NTA column (GE, USA) according to the purification protocol. The purified enzyme was pooled and desalted with a Hitrap desalting column (GE, USA), and then detected and quantified by SDS-PAGE and the Bradford method, respectively.

### 3.3. Mutant Library Construction for FLS

The site-directed mutagenesis libraries were constructed by the QuickChange method where mutagenic primers (Table S2) were used for amplification the entire recombinant plasmid pET28a-FLS containing the wild-type FLS gene [43]. The PCR reaction mixture (25 μL) contained plasmid template (10 ng), mutagenic primers (10 μM, 1.25 μL of each), 5× Q5 reaction buffer (5 μL), dNTPs (10 mM each, 0.5 μL) and Q5 DNA polymerase (0.25 μL). After PCR amplification, the restriction endonuclease *Dpn*I was used to completely digest the template plasmid pET28a-FLS in the PCR product. A total of 10 μL of digestion product was transformed into chemically competent *E. coli* BL21 (DE3) cells to create the relevant mutants. The sequences of positive variants were verified by DNA sequencing at Sangon Biotech Co., Ltd. (Shanghai, China).

### 3.4. Preliminary Screening of Mutant Library

At least 100 bacterial colonies of each site (statistically covering 95% of all 20 natural amino acids) generated by using site-directed saturation mutagenesis were picked out and inoculated into 96 deep-well plates, which contained 1 mL liquid LB broth per well (with 50 μg/mL kanamycin). After shaking incubation at 37 °C overnight, fifty microliters of the cultures were transferred to fresh 96-well plates containing 1 mL of fresh LB broth per well (with 50 μg/mL kanamycin) and incubated at 37 °C for 3 h. Subsequently, the inducer (0.5 mM IPTG) was supplemented into the cultures incubated at 18 °C for 24 h for expression of the target protein, and then the cells harvested by centrifugation were used to perform the screening of positive variants. The whole-cell-based screening method was performed by adding 1 mL reaction mixture (100 mM phosphate buffer, 100 mM acetaldehyde, 0.1 mM TPP, 1 mM Mg^2+^, pH 8.0) into deep-well plates and shaking at 180 rpm and 30 °C for 6 h. The product acetoin in the reaction supernatant was quantified by using the Voges–Proskauer (VP) test. Briefly, diluted reaction supernatant (0.3 mL), 0.5% creatine (0.3 mL), 5% alpha-naphthol (0.3 mL) and 5% NaOH (0.3 mL) were sequentially mixed together, and then reacted at 30 °C for 60 min. The OD_520_ of the reaction sample was measured by using a spectrophotometer, and the acetoin concentration was obtained from the calibration curve.

### 3.5. Substrate Tolerance Analysis of Beneficial Variants

The beneficial single-point and combinatorial variants were first selected to evaluate their substrate tolerance by using whole-cell catalysis, which was performed in a 1 mL reaction mixture containing 100 mM phosphate buffer (pH 8.0), 40 mg wet cell weight (WCW), 100–1000 mM acetaldehyde, 0.1 mM TPP and 1 mM Mg^2+^. Furthermore, the best mutational enzyme was purified and used to investigate its substrate tolerance in a 0.5 mL reaction mixture containing 100 mM phosphate buffer (pH 8.0), 100 μg purified enzyme, 0.5–4.0 M acetaldehyde, 0.1 mM TPP and 1 mM Mg^2+^. These reactions were conducted at 30 °C for 6 h in a rotary shaker with 180 rpm, and the acetoin concentration was quantified by using the VP test as described above.

### 3.6. Enzyme Activity and Kinetic Parameter Assays

The enzymatic reactions of FLS and its variants were performed with 2–600 mM acetaldehyde, 0.1 mM TPP, 1 mM Mg^2+^ and 30 μg purified enzyme in 500 μL phosphate buffer (100 mM, pH 8.0). The concentration of acetoin was measured by the VP test as described above. One unit of FLS activity (U) was defined as the amount of enzyme required to produce 1 μmol acetoin in 1 min. The values of the kinetic parameters (*K*_m_, *k*_cat_ and *k*_cat_/*K*_m_) were determined through the nonlinear regression fitting of the Michaelis–Menten equation. All assays were performed in triplicate.

### 3.7. Fed-Batch Synthesis of 2,3-BD from Ethanol and GC Analysis

A crude enzyme cocktail of 2.5 mg/mL EtDH:D46G, 5 mg/mL FLS:I28V/L482E, 2.5 mg/mL BDH:S199A and 2.5 mg/mL NOX was used for converting ethanol to 2,3-BD, as shown in Figure 1. The reaction was executed in a reaction mixture (initial volume of 5 mL) containing 1 mM NAD^+^, 1 mM NADP^+^, 5 mM Mg^2+^, 1 mM DTT, 0.5 mM TPP, 20% DMSO and 0.5 M ethanol with HEPES buffer (50 mM, pH 8.0) at 30 °C. The fed-batch experiment was carried out by feeding ethanol to a final concentration of 0.5 M into the reaction mixture every 3 h, and the amount of supplemented ethanol was 2.5 M in total. The reaction mixtures were detected using a gas chromatograph system as previously described [27].

## 4. Conclusions

In the present study, we obtained the variant FLS:I28V/L482E with significantly improved catalytic efficiency and acetaldehyde tolerance by engineering binding pockets and substrate tunnels. Then, 1.39 M (124.83 g/L) of 2,3-BD from low-cost ethanol with excellent productivity and yield values of 5.94 g/L/h and 92.7%, respectively, was obtained by using a modified four-enzyme synthetic biosystem combined with the fed-batch strategy. As far as we know, these results represent the highest titer, productivity and yield values for 2,3-BD synthesis by enzymatic approaches. Accordingly, our work opens a promising window for the cost-effective, sustainable and green upgrading of ethanol.

## Figures and Tables

**Table 1:** Kinetic parameters of WT FLS and its variants.

Enzyme	*K*_m_ (mM) ^a^	*k*_cat_ (s^−1^) ^a^	*k*_cat_/*K*_m_(s^−1^ M^−1^)	Ref.
FLS	58.46 ± 2.32	0.45 ± 0.04	7.69	This study
FLS:I28V	81.95 ± 3.61	3.33 ± 0.21	40.59	This study
FLS:L482E	50.95 ± 3.22	0.49 ± 0.06	9.66	This study
FLS:F484A	53.28 ± 2.52	1.74 ± 0.12	32.56	This study
FLS:I28V/L482E	375.55 ± 6.84	8.76 ± 0.13	23.34	This study
FLS:L482S	47.45 ± 1.20	0.63 ± 0.01	13.30	[27]

^a^ Data from three separate experiments are stated as the mean ± SD (*n* = 3).

**Table 2:** Summary of 2,3-BD production by enzymatic catalysis.

Enzyme	Method	Substrate	2,3-BD (g/L)	Ref.
The lysate from *E. coli* expressing ALS/ALDC/BDH	Cell-free	Glucose	82.00	[10]
BDH, NOX and Vitreoscilla hemoglobin	Whole-cell	Acetoin	38.41	[11]
*E. coli* expressing BDH	Whole-cell	Diacetyl	26.80	[13]
*Bacillus subtilis* expressing acetoin reductase and NOX	Whole-cell	Acetoin	74.50	[14]
The lysates from *E. coli* expressing ALS/ALDC/BDH and *cyanobacteria*	Cell-free	Starch	13.52	[42]
EtDH:D46G, FLS:I28V/L482E, NOX and BDH:S199A	Cell-free	Ethanol	124.83	This study

## Data Availability

The data presented in this study are available on request from the corresponding author. The data used to support the findings of this study can be made available by the corresponding author upon request.

## Supplementary Materials

The following supporting information can be downloaded at https://www.mdpi.com/article/10.3390/molecules29163934/s1: Figure S1: Screening of the variants of H26, T73, and N419 surrounding the substrate binding pocket of FLS and the wild-type FLS was used as control; Figure S2: SDS-PAGE analysis of the purified I28V/L482E variant; Figure S3: The effects of different acetaldehyde concentrations on the stability of the I28V/L482E variant; Figure S4: SDS-PAGE analysis of purified FLS and its variants; Figure S5: Substrate tunnel analysis of wild FLS and its variant I28V/L482E by Caver 3.0; Table S1: In silico analysis of substrate entrance tunnel by CAVER 3.0; Table S2: Primers used in this study.
