# Supplementary material for: Efficient 2,3-Butanediol Production from Ethanol by a Modified Four-Enzyme Synthetic Biosystem
Source: Molecules. 2024 Aug 20;29(16):3934. doi: 10.3390/molecules29163934 (PMC11357561; doi:10.3390/molecules29163934)
Supplement: Supplementary file 1 [file molecules-29-03934-s001.zip › molecules-3151157-supplementary.pdf]

Efficient 2,3-Butanediol Production from Ethanol by a Modified Four-Enzyme Synthetic Biosystem

Jiming Zhang [1,†], Hui Lin [2,†], Chaosong Zheng [3], Bin Yang [3], Miao Liang [3], Yi Lin [1,*] and Liaoyuan Zhang [3,*]

[1] College of Chemical Engineering, Huaqiao University, Xiamen 361021, China; jemeza@126.com
[2] Institute of Edible Fungi, Fujian Academy of Agricultural Sciences, Fuzhou 350012, China; 2170517002@fafu.edu.cn
[3] College of Life Sciences, Fujian Agriculture and Forestry University, Fuzhou 350002, China; 52305043027@fafu.edu.cn (C.Z.); lmiaojy@163.com (M.L.)
* Correspondence: lyhxm@hqu.edu.cn (Y.L.); zliaoyuan@126.com (L.Z.); Tel.: +86-591-83789492 (L.Z.); Fax: +86-591-83789121 (L.Z.)
† These authors contribute equally to this work.

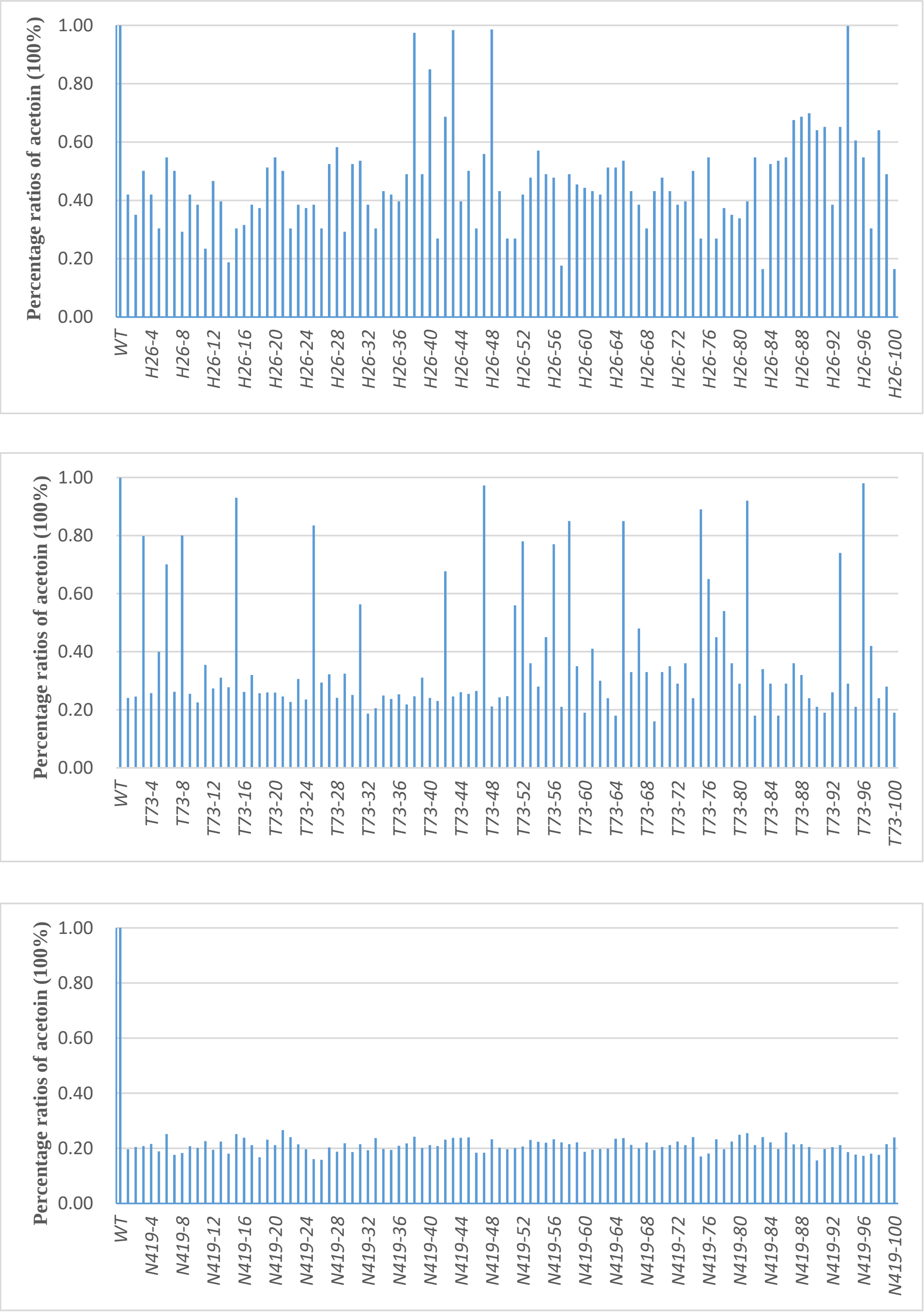

Figure S1. Screening of the variants of H26, T73, and N419 surrounding the substrate binding pocket of FLS and the wild-type FLS was used as control. 100 bacterial colonies of each site (statistically cover 95% of all 20 natural amino acids) generated by using site-directed saturation mutagenesis. Percentage ratios were calculated based on acetoin concentration by wild type FLS.

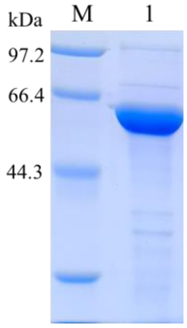

Figure S2. SDS-PAGE analysis of the purified I28V/L482E variant. M: Protein Marker (97.2 KDa, 66.4 KDa, 44.3 KDa, 29.0 KDa); 1: Purified I28V/L482E variant.

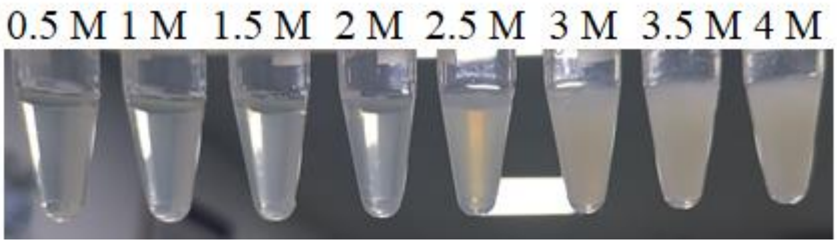

Figure S3. The effects of different acetaldehyde concentrations on the stability of the I28V/L482E variant.

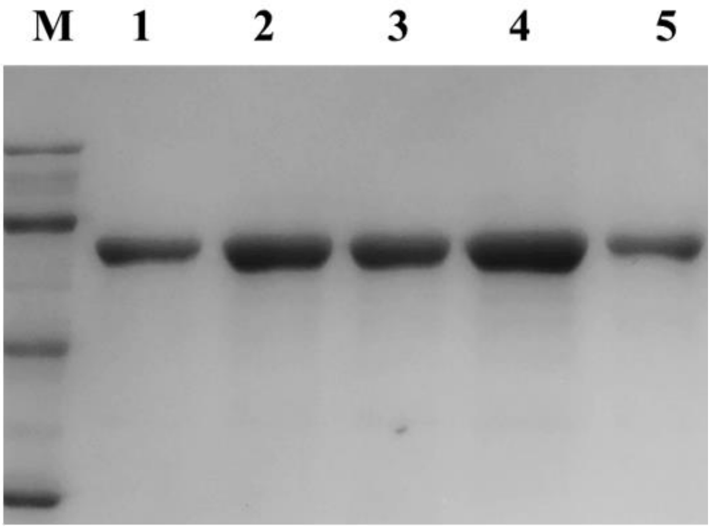

Figure S4. SDS-PAGE analysis of purified FLS and its variants. Lane 1: Wild-type FLS; Lane 2: FLS:I28V; Lane 3: FLS:L482E; Lane 4: FLS:F482A; Lane 5: FLS:I28V/L482E; M: Protein Marker (97.2 KDa, 66.4 KDa, 44.3 KDa, 29.0 KDa).

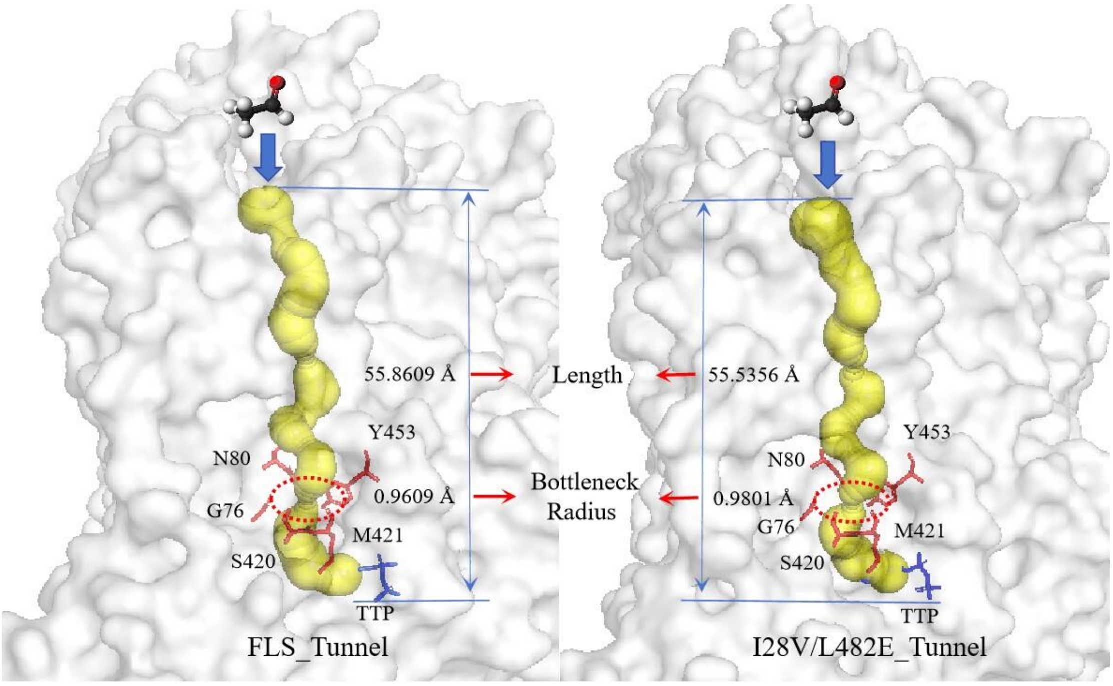

Figure S5. Substrate tunnel analysis of wild FLS and its variant I28V/L482E by Caver 3.0. The variant I28V/L482E led to increased bottleneck radius and reduced length of substrate entrance tunnel compared to wild FLS

Table S1. In silico analysis of substrate entrance tunnel by CAVER 3.0.

Enzyme	Throughput	Bottleneck radius /Å	Length /Å	Curvature
Wild FLS	0.1920	0.9609	55.8609	1.3869
I28V/L482E	0.2090	0.9801	55.5356	1.3768

Table S2. Primers used in this study.

**Primers**	**Sequence (5'-3')**	**Mutation site**
L482F	CCAAAGCTGGGGGTGGACANNKCATTTCCAGCAATTG	L482
L482R	CAATTGCTGGAAATGMNNTGTCCACCCCCAGCTTTGG	L482
T111F	TGATGAAACCAACNNKTTGCAGGCGGGGATTGATCAG	T111
T111R	TCCCCGCCTGCAAMNNGTTGGTTTCATCATCACGCAG	T111
W163F	GCTGGATCTGCCGNNKGATATTCTGATGAACCAGATT	W163
W163R	TCATCAGAATATCMNNCGGCAGATCCAGCAACACCGG	W163
L282F	CTTTGGCNNKAACACCGGGCATGGATCTG	L282
L282R	CGGTGTTMNNGCCAAAGCGCGCCCCCAG	L282
H483F	GGGGTGGACATTGNNKTTCCAGCAATTGGCCGTCGGC	H483
H483R	CCAATTGCTGGAAMNNCAATGTCCACCCCCAGCTTTG	H483
F484F	GACATTGCATNNKCAGCAATTGGCCGTCGGC	F484
F484R	GCTGMNNATGCAATGTCCACCCCCAG	F484
A488F	TTTCCAGCAATTGNNKGTCGGCCCCAATCGCGTGACG	A488
A488R	GATTGGGGCCGACMNNCAATTGCTGGAAATGCAATGT	A488
E553F	GATCCCGCCCGAANNKCTCATTCTGATCGGCATGGAC	E553
E553R	CGATCAGAATGAGMNNTTCGGGCGGGATCGGATCGAG	E553
L556F	CGAAGAACTCATTNNKATCGGCATGGACCCCTTCGCA	L556
L556R	GGTCCATGCCGATMNNAATGAGTTCTTCGGGCGGGAT	L556
I557F	TCATTCTGNNKGGCATGGACCCCTTCG	I557
I557R	TCCATGCCMNNCAGAATGAGTTCTTCG	I557
G27F	GCCTGCACNNKATTCATATCGATACGATTTTTCAAGC	G27
G27R	ATGAATMNNGTGCAGGCCGAACAGATGTT	G27
I28F	TCTGTTCGGCCTGCACGGCNNKCATATCGATACGA	I28
I28R	TCGTATCGATATGMNNGCCGTGCAGGCCGAACAGA	I28
L112F	TGAAACCAACACGNNKCAGGCGGGGATTGATCAGGTC	L112
L112R	CAATCCCCGCCTGMNNCGTGTTGGTTTCATCATCACG	L112
W480F	TGGGGGNNKACATTGCATTTCCAGCAATTGG	W480
W480R	TGCAATGTMNNCCCCCAGCTTTGGTTGTTC	W480
T481F	GGGGTGGNNKTTGCATTTCCAGCAATTGGC	T481
T481R	AATGCAAMNNCCACCCCCAGCTTTGGTT	T481
H26F	TCGGCCTGNNKGGCATTCATATCGATACGATTTTTC	H26
H26R	AATGCCMNNCAGGCCGAACAGATGTTCGA	H26
T73F	TGGCGCTGGTCNNKGCGGGCGGGGGATTTACC	T73
T73R	CGCMNNGACCAGCGCCACGCCCAG	T73
N419F	ATCTANNKTCGATGGGCGTGGGCTTC	N419
N419R	GCCCATCGAMNNTAGATAGCCGTGGCAGAGAAAAC	N419

Full-length EtDH:D46G

ATGACCCATCTGAATATTGCTAATCGCGTGGATAGTTTTTTTATTCCGTGTGTTACCCT
GTTTGGCCCGGGTTGCGCACGTGAAACCGGCGCCCGTGCACGTAGTCTGGGCGCACG
TAAAGCCCTGATTGTTACCGGTGCTGGCCTGCATAAAATGGGCCTGTCAGAAGTTGTT
GCAGGTCATATTCGTGAAGCTGGTCTGCAAGCAGTGATTTTTCCGGGCGCTGAACCGA
ATCCGACCGATGTTAATGTGCATGATGGTGTGAAACTGTTTGAACGTGAAGAATGTG
ATTTTATTGTGTCTCTGGGTGGCGGTAGCAGCCATGATTGCGCTAAAGGCATTGGCCT
GGTTACCGCGGGTGGCGGCCATATTCGCGATTATGAAGGTATTGATAAAAGTACCGT
GCCGATGACCCCGCTGATTTCAATTAATACCACCGCTGGTACAGCCGCCGAAATGAC
CCGCTTTTGTATTATTACCAATTCTAGTAATCATGTTAAAATGGCAATTGTTGATTGGC
GTTGCACCCCGCTGATTGCGATTGATGACCCTAGTCTGATGGTGGCAATGCCGCCGGC
GCTGACCGCGGCAACCGGCATGGATGCTCTGACCCATGCAATTGAAGCGTATGTGTC
AACCGCAGCCACCCCGATTACCGATGCGTGTGCAGAAAAAGCGATTGTGCTGATTGC
GGAATGGCTGCCGAAAGCGGTTGCGAATGGCGATAGCATGGAAGCCCGTGCGGCTAT
GTGCTATGCACAGTATCTGGCAGGCATGGCGTTTAATAATGCATCACTGGGTTATGTG
CACGCTATGGCACATCAGCTGGGCGGCTTTTATAATCTGCCGCATGGTGTGTGCAATG
CAATTCTGCTGCCGCATGTGTCAGAATTTAATCTGATTGCCGCACCGGAACGCTATGC
ACGCATTGCCGAACTGCTGGGTGAAAATATTGGTGGTCTGTCTGCTCATGATGCGGCG
AAAGCTGCTGTTAGCGCGATTCGCACCCTGTCTACCTCTATTGGTATTCCGGCTGGTC
TGGCGGGTCTGGGTGTTAAAGCCGATGACCATGAAGTTATGGCCAGCAATGCTCAGA
AAGATGCTTGTATGCTGACCAATCCGCGCAAAGCCACCCTGGCCCAGGTTATGGCGA
TTTTTGCTGCCGCCATGTAA

Full-length NOX

ATGAAGATTCTTGTCATTGGTGCTACCCATGCCGGTACATTTGCAACCCAGCAGATCC
TAACCGACCATCCAGATGCAGAGGTTACTGTCTACGAACGCAATAACAACCTGTCCTT
CCTCTCGTGCGGCATTGCCTTGTGGGTTGGTGATCATGTCAGTGACCCGGATAAAATG
TTCTATTCCAGTCCCGAAGCACTCGCTAAACTCGGTGCTAATATGCAAATGGAACATG
ATGTGCTCAATATTGATCCAGCAACTAAAACAGTTGAAGTCAAGGATCTAAAAACCG
GAACCGTTACTACCGATACTTATGACAAATTAGTCTACACAACCGGATCGACGCCAA
TCATTCCAAATATTCCCGGTATCCACGATTCAAACGTCTACTTATGCAAAAATTGGTC
CGACGCCAAGACGCTAAAAGATCTGGCCCCGTCCATTAAAAGCGCCATTGTCATCGG
TGCAGGCTACATCGGTGCAGAATTAGCCGAACAATTTGCGTTAACCGACAAAGAAGT
CACGTTAATCGATGGACTTCCACGGGTTTTGGCGAAAAACTTTGACGCCACTATCACG
GATCGCGTTGAAAAGCTTTACACCGATCACGGGGTTCACTTGGCACTCAATGAGATG
GTTACCGAGTTCGCACAAGCTGATCAGGGTATCAAGGTTACAACCAATAAAGGCGAC
TATACCGCGGATATTGCAATTTTATGTACCGGCTTCCGTCCGAACACGGATCTGCTAA
AGGACCATCTGGACACCCTGCCTAATGGCGCTGTCATAACAAATGCATATATGCAGA
CCAGTGACCCCGACATTTTCGCTGCTGGTGATACCGCTACCGTCCACTATAATCCGAC
TGGCAAAAATGACTACATCCCGCTTGCGACCAACGCAGTCCGTCAAGGCATTCTTGTT
GGTAAAAATATCATGACCCCCACGGAAAAATACCTGGGAACACAATCTAGCTCGGCC
GTTGAACTTTTTGATCACGCCATTGCGGCAAGCGGCCTAACGGTGGAAGGCGCTCAC
ACACGTGGACTTGAGCTTGATAGTGTCACGATCGAACAGGATTATCGCCCCGATTTCA
TGTTAACCACAACGCCGGTGCTCTGCAGCCTGACATGGGATCCCAAGACGCATGAAG
TTAAAGGAGGTGCCTTTTTCTCCAAGCACGATATCAGCCAAAGCGCTAATGTCATTTC
GCTTGCGATCCAGACCCACATGACGATCGAAACACTTGCGATGGTTGACATGCTCTTC
CAACCTAACTTCGATCAGCCGATTAACTGGGTAAATGCCGTGGCTATGGCGGCAGTT
GACAAGGCTAAAAAGAAGCCGACAACACCGGTAGCCTAA

Full-length FLS

ATGGCGATGATTACAGGCGGCGAACTGGTTGTTCGCACCCTAATAAAGGCTGGGGTC
GAACATCTGTTCGGCCTGCACGGCATTCATATCGATACGATTTTTCAAGCCTGTCTCG
ATCATGATGTGCCGATCATCGACACCCGCCATGAGGCCGCCGCAGGGCATGCGGCCG
AGGGCTATGCCCGCGCTGGCGCCAAGCTGGGCGTGGCGCTGGTCACGGCGGGCGGGG
GATTTACCAATGCGGTCACGCCCATTGCCAACGCTCGTACCGATCGCACGCCGGTGCT
CTTCCTCACCGGATCGGGCGCGCTGCGTGATGATGAAACCAACACGTTGCAGGCGGG
GATTGATCAGGTCGCCATGGCGGCGCCCATTACCAAATGGGCGCATCGGGTGATGGC
AACCGAGCATATCCCACGGCTGGTGATGCAGGCGATCCGCGCCGCGTTGAGCGCGCC
ACGCGGGCCGGTGTTGCTGGATCTGCCGTGGGATATTCTGATGAACCAGATTGATGA
GGATAGCGTCATTATCCCCGATCTGGTCTTGTCCGCGCATGGGGCCCATCCCGACCCT
GCCGATCTGGATCAGGCTCTCGCGCTTTTGCGCAAGGCGGAGCGGCCGGTCATCGTG
CTCGGCTCAGAAGCCTCGCGGACAGCGCGCAAGACGGCGCTTAGCGCCTTCGTGGCG
GCGACTGGCGTGCCGGTGTTTGCCGATTATGAAGGGCTAAGCATGCTCTCGGGGCTG
CCCGATGCTATGCGGGGCGGGCTGGTGCAAAACCTCTATTCTTTTGCCAAAGCCGATG
CCGCGCCAGATCTCGTGCTGATGCTGGGGGCGCGCTTTGGCCTTAACACCGGGCATG
GATCTGGGCAGTTGATCCCCCATAGCGCGCAGGTCATTCAGGTCGACCCTGATGCCTG
CGAGCTGGGACGCCTGCAGGGCATCGCTCTGGGCATTGTGGCCGATGTGGGTGGGAC
CATCGAGGCTTTGGCGCAGGCCACCGCGCAAGATGCGGCTTGGCCGGATCGCGGCGA
CTGGTGCGCCAAAGTGACGGATCTGGCGCAAGAGCGCTATGCCAGCATCGCTGCGAA
ATCGAGCAGCGAGCATGCGCTCCACCCCTTTCACGCCTCGCAGGTCATTGCCAAACAC
GTCGATGCAGGGGTGACGGTGGTAGCGGATGGTGGCCTGACCTATCTCTGGCTGTCC
GAAGTGATGAGCCGCGTGAAACCCGGCGGTTTTCTCTGCCACGGCTATCTAAACTCG
ATGGGCGTGGGCTTCGGCACGGCGCTGGGCGCGCAAGTGGCCGATCTTGAAGCAGGC
CGCCGCACGATCCTTGTGACCGGCGATGGCTCGGTGGGCTATAGCATCGGTGAATTTG
ATACGCTGGTGCGCAAACAATTGCCGCTGATCGTCATCATCATGAACAACCAAAGCT
GGGGGTGGACATTGCATTTCCAGCAATTGGCCGTCGGCCCCAATCGCGTGACGGGCA
CCCGTTTGGAAAATGGCTCCTATCACGGGGTGGCCGCCGCCTTTGGCGCGGATGGCTA
TCATGTCGACAGTGTGGAGAGCTTTTCTGCGGCTCTGGCCCAAGCGCTCGCCCATAAT
CGCCCCGCCTGCATCAATGTCGCGGTCGCGCTCGATCCGATCCCGCCCGAAGAACTCA
TTCTGATCGGCATGGACCCCTTCGCATGA

Full-length BDH:S199A

ATGAAAGGTTTTGCAATGTTAGGTATTAACAAATTAGGATGGATTGAAAAGAAAAAC
CCAGTGCCAGGTCCTTATGATGCGATTGTACATCCTCTAGCTGTATCCCCATGTACAT
CAGATATACATACGGTTTTTGAAGGAGCACTTGGTAATAGGGAAAATATGATTTTAG
GCCATGAAGCTGTAGGTGAAATAGCCGAAGTTGGCAGCGAAGTTAAAGATTTTAAAG
TTGGCGATAGAGTTATCGTACCATGCACAACACCTGACTGGAGATCTTTAGAAGTCCA
AGCTGGTTTTCAGCAGCATTCAAACGGTATGCTTGCAGGATGGAAGTTTTCCAATTTT
AAAGATGGTGTATTTGCAGATTACTTTCATGTAAACGATGCAGATATGAATCTTGCCA
TACTCCCAGATGAAATACCTTTAGAAAGTGCAGTTATGATGACAGACATGATGACTA
CTGGTTTTCATGGAGCAGAACTTGCAGACATAAAAATGGGCTCCAGCGTTGTAGTAA
TTGGTATAGGAGCTGTTGGATTAATGGGAATAGCCGGTTCCAAACTTCGAGGAGCAG
GCAGAATTATCGGTGTTGGAGCCAGACCTGTTTGTGTTGAAACAGCTAAATTTTATGG
AGCAACTGATATTGTAAATTATAAAAATGGTGATATAGTTGAACAAATCATGGACTT
AACTCATGGTAAAGGTGTAGACCGTGTAATCATGGCAGGCGGTGGTGCTGAAACACT
AGCACAAGCAGTAACTATGGTTAAACCTGGCGGCGTAATTTCTAACATCAACTACCA
TGGAAGCGGTGATACTTTACCAATACCTCGTGTTCAATGGGGCTGCGGCATGGCTCAC
AAAACTATAAGAGGAGGATTATGCCCCGGCGGACGTCTTAGAATGGAAATGCTAAGA
GATCTTGTTCTATATAAACGTGTTGATTTGAGTAAACTTGTTACTCATGTATTTGATGG
TGCAGAAAATATTGAAAAGGCCCTTTTGCTTATGAAAAATAAGCCAAAAGATTTAAT
TAAATCAGTAGTTACATTCTAA
